# Selective Integration of Social Feedback Promotes a Stable and Positively Biased Self‐Concept

**DOI:** 10.1111/sjop.13113

**Published:** 2025-04-01

**Authors:** Josué García‐Arch, Marc Sabio‐Albert, Lluis Fuentemilla

**Affiliations:** ^1^ Department of Cognition, Development and Education Psychology, Faculty of Psychology University of Barcelona Barcelona Spain; ^2^ Institute of Neuroscience (UBNeuro) University of Barcelona Barcelona Spain; ^3^ Bellvitge Institute for Biomedical Research Barcelona Spain

**Keywords:** belief updating, self‐concept, self‐concept clarity, social feedback

## Abstract

Understanding self‐concept dynamics is crucial given its generalized impact on our well‐being. However, how we integrate information into our self‐representations to promote a positively biased, yet progressively stable self‐concept is a question that remains unanswered. In a series of four experiments, we refined a belief updating task to investigate how participants integrate social feedback depending on its valence and self‐congruence. Experiment 1 indicated that the lack of control of an initial positive bias in participants self‐concept might have masked valence and congruence effects in recent works. After implementing methodological adjustments (Experiments 2 and 3) we found that the integration of social feedback was strongly driven by feedback self‐congruence and moderately driven by feedback valence. By synthesizing insights from social, personality, and cognitive psychology, this study advances the understanding of self‐concept dynamics during social feedback processing. Our conceptual and methodological advancements offer a new lens for reinterpreting previous findings.

**Summary**

- Self‐concept updates strongly prioritize stability by selectively integrating self‐congruent over self‐incongruent social feedback.
- Positive feedback is moderately favored, reinforcing a positively biased self‐concept.
- These findings bridge theoretical gaps between self‐enhancement and self‐verification perspectives in social psychology within the context of belief updating.
- Methodological improvements developed here allow precise separation of positivity and congruence effects in self‐concept updating.

## 1 Introduction

Our self‐concept contains information about our personality and life experiences that help us define who we are and what we can expect from ourselves (Conway et al. 2004; Epstein 1973; Grilli and Verfaellie 2014; Martinelli et al. 2013; Rathbone et al. 2008). However, the self‐concept is not static, and the formation of self‐representations is a continuous process (Conway 2005; Manzi et al. 2010; Markus and Wurf 1986). This process of construction and revision of self‐representations is particularly influenced by social feedback (Crone et al. 2022; Reitz et al. 2014; Rodman et al. 2017). During social interactions, we are often confronted with feedback about our attributes, which serves us to shape the way we see ourselves. Numerous studies suggest that we achieve and maintain a positive self‐concept by seeking positive evaluations and selectively integrating them into our self‐representations (Alicke and Sedikides 2009; Hepper et al. 2011; Korn et al. 2012; Taylor et al. 1988). From this perspective, the self‐concept is portrayed as a motivated system that selectively incorporates information that maximizes its positivity, even at the expense of accuracy (Alicke and Sedikides 2009; Sedikides and Alicke 2019). In contrast, research also suggests that our self‐representations are embedded in a highly organized self‐knowledge system that protects the self‐concept from long‐term stability violations (Conway 2005; Conway et al. 2004; Conway and Pleydell‐Pearce 2000; Grilli 2017; Rathbone et al. 2008). Accordingly, there is also evidence that we tend to reject self‐discrepant feedback (Swann Jr. and Brooks 2012; Swann et al. 1984; Swann and Hill 1982) and strive to receive social inputs that are consistent with our self‐representations, even if they are negative (Swann and Buhrmester 2012; Swann, Tafarodi, et al. 1992). Understanding self‐concept stability‐malleability dynamics is crucial, given its generalized impact on our cognition, behavior, and affect (Beck et al. 1990; Libby and Eibach 2002; Marsh and Martin 2011). However, how we incorporate self‐relevant information to promote a positively biased but progressively stable self‐concept is a question that remains unanswered.

On one side of these opposing views, there is a broad consensus that we are primarily motivated to achieve and maintain a positively biased self‐concept. For example, we often believe we possess more favorable traits than others and tend to overestimate our abilities and positive attributes (Dunning et al. 2003; Preuss and Alicke 2009; Zell et al. 2020). The impact of our self‐concept on our well‐being is critical and pervasive, and it has been suggested that the motive for achieving and preserving positive self‐representations is inherent in psychologically healthy adults (Hepper et al. 2010). Extensive research has also suggested that we are highly skilled in processing information in favor of ourselves (Boseovski 2010; Taylor et al. 1988). In social interactions, we strive to receive positive feedback and prefer interaction partners who are more likely to provide it (Hepper et al. 2010). We overestimate the likelihood of receiving positive evaluations from others and devote efforts to restore positive self‐representations after facing threatening feedback (Rodman et al. 2017). Recently, a growing body of experimental research has suggested that we achieve a positively biased self‐concept through the selective incorporation of positive over negative information. This research leverages robust belief updating paradigms to explore how new information is incorporated into prior beliefs. Findings in this field have revealed that when facing new self‐relevant social feedback, negative inputs tend to be largely disregarded, whereas positive feedback prompts a shift in self‐representations toward a feedback‐consistent direction (Elder et al. 2022; Korn et al. 2012, 2014). This feedback‐based valence asymmetry in updating the self‐concept has provided experimental results with large effect sizes, and it has been shown to be cross‐culturally invariant (Korn et al. 2014) and important for psychological well‐being (Korn et al. 2016). These studies suggest that our bias toward positive feedback extends beyond mere preference or active seeking behavior; it reveals that we selectively utilize positive feedback to shape and enhance our self‐representations.

Although the view of the self‐concept as a system that tries to maximize its positivity is widely accepted, this conceptualization overlooks important features of self‐representations that have been extensively studied in other fields of research in psychology. For example, from a cognitive perspective, self‐representations are conceived as an endurable and well‐grounded form of personal semantic knowledge embedded in a highly structured system of autobiographical information (Conway 2005; Haslam et al. 2011; Klein 2010; Rathbone and Moulin 2014; Renoult et al. 2012). There is evidence that our self‐representations are formed, supported, and contextualized by a wide range of episodic memories we have encoded from extended, repeated, and self‐defining events (Rathbone and Conway 2009; Conway 2005; Rathbone et al. 2008). This highly organized set of self‐relevant information provides stability and coherence and allows us to remember and anticipate trait‐congruent experiences and select adaptive behaviors (Conway et al. 2004). From this view, our need to sustain self‐concept stability and coherence operates as a powerful constraint that determines which upcoming external inputs would be encoded and remembered, favoring self‐congruent information (Conway 2005; Conway et al. 2004; Conway and Pleydell‐Pearce 2000). Accordingly, social psychology studies have revealed that in social interactions we strive to receive feedback that is congruent with our self‐beliefs (Robinson and Smith‐Lovin 1992; Swann Jr. and Brooks 2012). Perhaps the most remarkable example is that individuals with positive self‐concepts are inclined to seek out and prefer positive feedback, while those with negative self‐concepts tend to search for negative feedback (Kwang and Swann 2010; Swann, Tafarodi, et al. 1992). This phenomenon has lent support to the notion that adding confirming evidence to our self‐beliefs might be more important than receiving positive evaluations (Swann Jr. and Brooks 2012; Swann et al. 1989). Similarly, research indicates that self‐discrepant social feedback prompts compensatory responses to protect our self‐concept (Swann and Hill 1982), and that negative feedback exerts a greater detrimental effect on our mood if it is self‐incongruent (van Schie et al. 2018). In sum, this research emphasizes that we seek a stabilized self‐concept because it helps us understand ourselves, select appropriate interaction partners, and predict our behavior and affect throughout life (Conway 2005; Steele 1988, Swann and Brooks, 2012). Therefore, these proposals cast doubt on the potential of social feedback to trigger changes in our self‐concept with the sole motivation of pursuing self‐enhancement.

This conflicting evidence leaves an important question unanswered. A key aspect to resolve is how we integrate new information from our environment into our self‐representations to promote a positively biased but progressively stable self‐concept. While research has provided some insights into the role of positivity seeking in self‐concept updating, separate research lines suggest that indiscriminate integration of positive (or negative) feedback might compromise self‐concept stability.

To illustrate the importance of integrating insights from these different research lines, consider the following examples. Imagine that we are asked about how sociable we are. Based on our knowledge and experiences, we might be certain that we would never use that trait to describe ourselves. However, we receive social feedback indicating that we are more sociable than we believe we are. A social evaluation suggesting that we should reconsider if we identify as a sociable person not only would be self‐incongruent, but it would also provide the means to see ourselves in a better light. Would that make us align with the social feedback received to increase the positivity of our self‐concept? Although accepting positive and incongruent feedback would bring us closer to considering socially desirable traits as our own, it might challenge the stability of our self‐concept. Accepting it and shifting our self‐evaluations in a feedback‐consistent direction may lead us to internalize a positive trait that has no actual correlate in our behavior. In the opposite scenario, we may recognize ourselves as someone who is sociable. Following a self‐evaluation of that characteristic, we receive a social assessment indicating that we should view ourselves as more sociable than we initially thought. Adjusting our self‐representation in a feedback‐consistent direction not only would increase our self‐concept positivity, but it would also strengthen the very notion that we are, indeed, sociable. This congruent‐like feedback experience is likely to have more chances of being integrated into our self‐concept, as it would add confirming (and positive) evidence to an already well‐grounded self‐representation. Note that the reverse is also true when receiving social evaluations suggesting that we are less sociable than we thought we were. In the case of considering “sociable” as self‐descriptive, shifting our self‐representations in a feedback‐consistent direction would conflict with the autobiographical evidence we have to support this self‐representation (Conway 2005; Conway et al. 2004). In turn, it would entail a loss of positivity in our self‐concept. This might be the perfect scenario to minimize, ignore, or even compensate for the feedback received. In contrast, if we consider sociable as non‐self‐descriptive, negative feedback would strengthen the notion that we have no evidence to believe we behave as a sociable person. Therefore, it might stabilize our self‐concept, provide evidence that we are accurate in our self‐judgments (Swann Jr. and Brooks 2012), and distance the possibility of having to internalize a new trait into our self‐concept with little to no evidence to draw on. In this case, there is no apparent reason to ignore the feedback received but the fact that being considered sociable is socially desirable (Anderson 1968). Note that, for negative traits, the same logic can be applied as in the examples above. Taken together, it is plausible to propose that the pursuit of positivity and the need for stability in our self‐concept can work in conjunction to shape a self‐concept that evolves toward an improved version while preserving its core content. This alignment would be in line with early (Swann et al. 1989) and up‐to‐date theoretical insights (Mokady and Reggev 2022).

In the present study, we aimed to orthogonalize and test the effect of both positivity and stability constraints on participants' integration of self‐relevant social feedback. In Experiment 1, our objective was to determine the extent to which the initial bias in participants' self‐concept would mask the effect of positivity‐seeking and stability‐seeking motives in updating the self‐concept. To further address this issue, in subsequent experiments (Experiments 2 and 3) we isolated the effects of feedback valence (positive vs. negative) and feedback self‐congruence (congruent vs. incongruent) and explored their potential as enduring constraints on self‐concept updating. Finally, we aimed to investigate the specificity of these effects by testing their generalizability to the updating of representations we hold about others (Experiment 4).

## 2 Experiment 1

### 2.1 Introduction

The isolated study of the seek for positivity and the need for self‐concept stability has produced apparently conflicting results. Although there are also works testing which one can better explain our social feedback choices (Kwang and Swann 2010), whether both motives play a role in the way we incorporate social evaluations in our self‐concept is a question that remains unanswered. In turn, the lack of consideration of both motives in the study of self‐concept updating might lead to an essential conceptual ambiguity. Available evidence has robustly suggested that when updating beliefs about our own traits we tend to integrate positive feedback and discard negative evaluations (Elder et al. 2022; Korn et al. 2012, 2014, 2016). However, given that self‐concepts are often characterized by a positive bias (i.e., we tend to perceive ourselves more favorably than unfavorably), the positive feedback participants receive in these studies may primarily be perceived as congruent feedback, whereas negative feedback may be incompatible with participants' current self‐representations. Similar concerns have already been pointed out in related literature (Swann Jr. and Brooks 2012).

In Experiment 1, we aimed to reproduce the valence‐dependent belief updating found in prior research to exemplify and quantify how participants' initial bias positive bias in their self‐evaluations might be masking the effect of positivity and stability constraints on self‐concept updating. This served as an initial step to develop an experimental paradigm that can effectively isolate the effects of feedback valence and feedback self‐congruence on participants' self‐concept updating (Experiment 2).

### 2.2 Methods

#### 2.2.1 Participants

Prior to the experiment, we conducted a power analysis using G*Power (Faul et al. 2007) to determine the required sample size. Power was determined for testing the main effect of feedback valence (positive vs. negative) on participants belief updating. We based the expected effect size (*η*_p_^2^) on prior research in this field, which has reported partial eta squared values above *η*_p_^2^ = 0.3 (e.g., Korn et al. 2012, 2014, 2016). We assumed a partial eta squared of 0.1 with a default correlation between measures of 0.5. Power analysis revealed that for an acceptable power of 0.8 (*α* = 0.05) we needed 20 participants, and we recruited 36 (undergraduate students, 23 female and 23 male, *M*_age_ = 23.22, SD_age_ = 3.94). Participants provided informed consent before their participation. The study protocol was approved by the ethics committee of the University of Barcelona (Institutional Review Board IRB00003099).

#### 2.2.2 Procedure

Participants took part in a two‐session online experiment on two consecutive days. The first session was administered via Qualtrics (www.qualtrics.com) and the second session via Pavlovia (www.pavlovia.org). The aim of the first session was to create a situation in which participants believed they would receive relevant social feedback during the second session. The second session consisted of performing the experimental task.

##### 2.2.2.1 First Session

In this session, participants were presented with three audio recordings of personality self‐descriptions embedded in a Qualtrics questionnaire. Participants were informed that these recordings were randomly selected from other members of the experimental sample. However, the voice clips were made by external collaborators who were not initially informed of the purpose of the study to maintain the authenticity of the recordings. After finishing their voice clips, the collaborators were informed about the study's objectives and provided their consent for their utilization. Each audio recording had a duration of approximately 6 min (min: 5.32, max: 6.73) and the order in which they were presented to the participants was randomly determined. After listening to each recording, participants were asked to evaluate the personality of the speaker by rating a list of 40 adjectives on a scale from 1 (i.e., the adjective does not fit with the description of the person I listened to) to 8 (i.e., the adjective fits perfectly with the person I listened to). Participants were then asked to record themselves describing their personality. They received detailed guidelines of how the audios should be made and they were told to use the three recordings presented previously as examples. The guideline consisted of 12 items randomly drawn from the HEXACO personality questionnaire (https://hexaco.org/; e.g., “I feel reasonably satisfied with myself overall”, “I rarely express my opinion in social meetings”, see Table S1). Participants were instructed to speak for at least 30–45 s about each statement, offering their degree of agreement with them and explaining the reason for their answer with examples or anecdotes. Once the recording was completed, participants attached it to the online questionnaire. Finally, participants were requested to complete the Beck Depression Inventory (BDI‐II), which score was used as an exclusion criterion. Following previous research (Garcia‐Arch et al. 2022; Kappes and Sharot 2019), participants that scored > 19 in the BDI were excluded from the data analysis. In the current experiment, one participant scored 22 and was excluded from further analysis. Participants who had missed more than 15% of the trials in any of the two experimental tasks (see below) were also excluded from the sample. Two participants met these later criteria and were excluded from further analysis.

##### 2.2.2.2 Second Session

**FIGURE 1:**
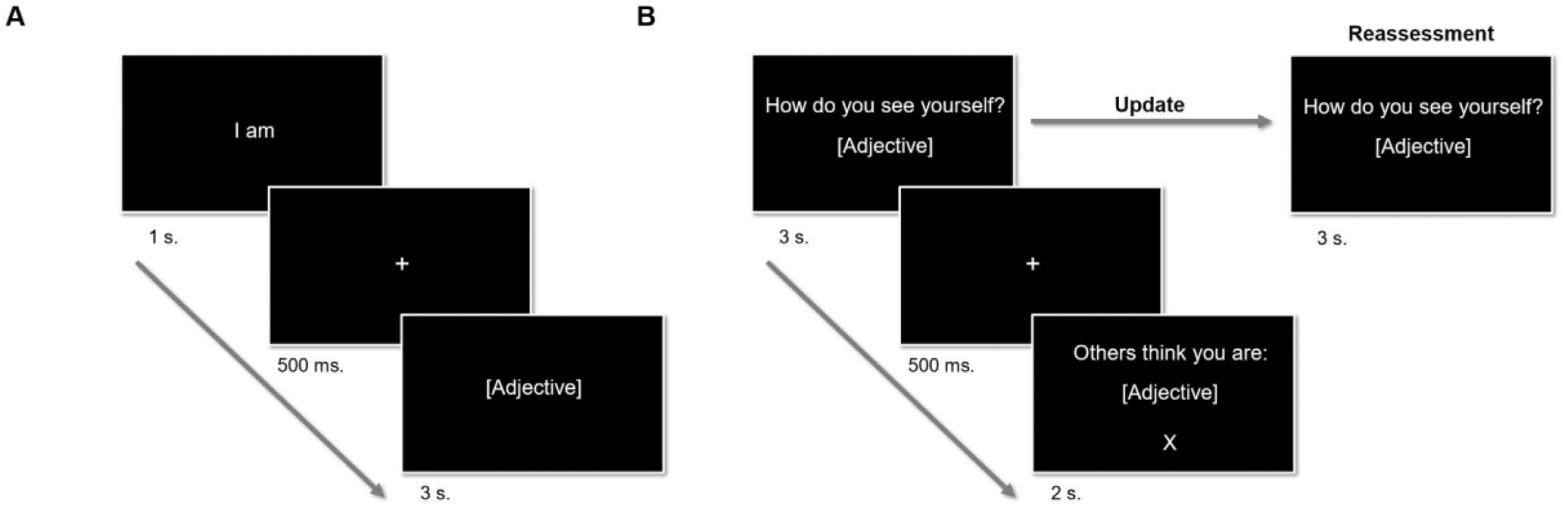
Experiment outline. Sentence verification task (A). During the task, participants were tasked with determining whether the adjectives presented in each sentence described themselves or not (e.g., “I am friendly”). The trial structure involved a sequence of events, starting with a fixation cross lasting 500 ms, followed by the sentence “I am” displayed for 1 s. After another 500 ms fixation cross, an adjective appeared on the screen (e.g., “friendly”) and remained visible for 3 s. Participants had 3 s to respond by using the left arrow (for “Yes”) or the right arrow (for “No”) on their computer keyboards. Belief updating task (B). In each trial, participants were presented with 1 of 40 trait adjectives together with the prompt “how do you see yourself?” and had to think about how much that trait applied to themselves and rate it on a Likert scale from 1 (this trait does not describe me at all) to 8 (this trait describes me perfectly; 3 s). After another fixation cross (500 ms) participants saw what they believed to be the mean rating that three other participants provided about them on that trait after listening to their audio recording (2 s). That rating, which served as a feedback rating, was a number ranging from 1.0 to 8.0 in steps of 0.5. As in prior studies (Korn et al. 2012), feedback ratings were pseudo‐randomly generated by the program during the experiment to produce a balanced number of trials in which participants received positive and negative feedback. After repeating this process for all adjectives, participants rated themselves again on all traits (re‐evaluation).

In the second session, participants performed two consecutive experimental tasks. First, participants underwent a sentence verification type task (Figure 1A), which has previously been used to study personal semantic memory and self‐representations (Renoult et al. 2012). This task consists of accepting and rejecting sentences by means of a dichotomous response, typically “Yes” versus “No”. In this task, participants were asked to decide whether the adjectives presented in each sentence represented themselves or not (e.g., “I am friendly”). The trial structure consisted of a fixation cross (500 ms) followed by the sentence “I am” (1 s). After another fixation cross (500 ms) an adjective appeared on the screen (e.g., “friendly”, 3 s). At that moment, they had 3 s to provide their responses using the left (Yes) and right (No) arrows on their computer keyboards.

Next, participants responded to a belief updating task that has been previously used to study the impact of positive and negative feedback on beliefs about one's own personality traits (Elder et al. 2022; Korn et al. 2012, 2014). In each trial, participants were presented with 1 of 40 trait adjectives together with the prompt “how do you see yourself?” and had to think about how much that trait applied to themselves and rate it on a Likert scale from 1 (this trait does not describe me at all) to 8 (this trait describes me perfectly; 3 s). After another fixation cross (500 ms) participants saw what they believed to be the mean rating that three other participants provided about them on that trait after listening to their audio recording (2 s). That rating, which served as a feedback rating, was a number ranging from 1.0 to 8.0 in steps of 0.5. As in prior studies, feedback ratings were pseudo‐randomly generated by the program during the experiment to produce a balanced number of trials in which participants received positive and negative feedback. After repeating this process for all adjectives, participants rated themselves again on all traits (reassessment). Once both tasks were completed, participants were asked to recall the feedback ratings (Likert scale from 1 to 8) they had received for each trait in a separate questionnaire.

##### 2.2.2.3 Main Measures

The main measures extracted from the experimental tasks were participants' categorical responses (Yes vs. No), pre‐ and post‐feedback self‐assessments (8 points Likert scale), valence of the feedback received (positive vs. negative), and feedback rating (8 points Likert scale, in steps of 0.5). Pre‐ and post‐feedback self‐assessments were used to compute the dependent variable of interest of the study, namely, update scores. Update scores represent the degree of change of initial beliefs after receiving social feedback. We computed this variable so that it could be interpreted as the degree of feedback‐consistent update (i.e., the degree to which a belief changes in the direction that the feedback suggests; Korn et al. 2012). Specifically, this measure was computed as follows: when the feedback received was lower than the self‐assessment, update scores were computed as self‐assessment(pre)–self‐assessment(post). When the feedback received was higher than the self‐assessment, update scores were equal to self‐assessment(post)–self‐assessment(pre). Note, that we allowed negative values for this variable, which denote that the participant has changed their self‐representations in the opposite direction to what the feedback suggested. Feedback valence was determined as follows: if a positive adjective received a feedback rating lower than the self‐rating or a negative adjective received a higher feedback rating than the self‐rating, the feedback was labeled as negative. If the feedback received for a positive adjective was higher than the self‐rating or the feedback for a negative adjective was lower than the self‐rating, feedback was labeled as positive. Categorical responses from the sentence verification task were used in combination with feedback valence to generate a measure of feedback self‐congruence. Feedback self‐congruence was operationalized as a two‐level factor (self‐congruent vs. self‐incongruent) that aimed to capture the extent to which the feedback received had the potential to either reinforce or conflict with participants' self‐representations. To illustrate this idea, imagine a participant who in the sentence verification task decides that the trait “sociable” does not apply to them. Subsequently, in the belief updating task when they have to provide a self‐rating on the same trait, they select a “3” (out of 8). In the case of receiving a score lower than 3 as (negative feedback), the feedback received would be supporting their notion that they are not a sociable person (self‐congruent feedback). In contrast, if they receive a score higher than 3 (positive feedback), the feedback received would be suggesting that they should reconsider the notion that they are not a sociable person (self‐incongruent feedback). As in most studies in related literature, we also computed a covariate aimed to capture the degree of mismatch between participants' self‐ratings and feedback ratings (i.e., numerical distance), namely, feedback discrepancy. This measure was computed for each participant as the average absolute difference between self‐ratings and feedback ratings. We introduced another measure to provide additional control to the analysis. This measure was intended to capture how much space within the scale participants had available for updating. We named this measure update space. The update space was computed as the average difference between participants' self‐assessments(pre) and the limit of the scale (8‐point Likert scale in our case) taking into account the direction suggested by the feedback. That is, if self‐assessment(pre) < feedback, then update space = 8‐self‐assessment(pre). If self‐assessment(pre) > feedback, then update space = self‐assessment(pre)–1.

##### 2.2.2.4 Stimuli

For this experiment, we randomly selected 20 positive and 20 negative adjectives from prior studies (Korn et al. 2012, 2014), which come from a widely used list of personality attributes (Anderson 1968). We asked a separate sample of participants (*n* = 42) to provide observability ratings on each adjective. Specifically, we asked participants to decide the extent to which each adjective would be discerned from a given individual by listening to a 6‐min personality description. We provided participants the specific guidelines those individuals would use to describe themselves. Participants provided those ratings using a Likert scale ranging from 1 (not observable at all) to 8 (very observable). We selected only those adjectives with an average observability rating above 4.5. The final sample of stimuli consisted of a list of 31 adjectives describing character traits (16 positive and 15 negative). To equate the number of adjectives between valence categories, we selected one negative adjective to complete the list based on its closeness to our inclusion criterion. Selected stimuli were classified as positive or negative according to their previously reported average desirability ratings (Anderson 1968) and prior classifications (Korn et al. 2012, 2014, 2016). For the final list of adjectives see Table S2. To check if this classification remained stable in our sample, we asked participants to provide desirability ratings for all the adjectives in the list. We then compared participants' average ratings of positive vs. negative adjectives by means of a paired *t*‐test. The Paired *t*‐test testing the difference between desirability ratings on positive versus negative adjectives showed that positive adjectives received significantly higher desirability ratings than negative adjectives (*M*_dif_ = 3.068, 95% CI [2.849, 3.286], *t*(33) = 28.578, *p* < 0.001; *d* = 4.974, 95% CI [3.338, 5.591]). In addition, we tested whether the desirability ratings of positive adjectives were above the mid‐point scale (4.5) by means of a one‐tailed one‐sample *t*‐test (against mu = 4.5). Finally, we tested whether the desirability ratings of negative adjectives were below the mid‐point scale (4.5). Desirability ratings of adjectives classified as positive showed to be significantly above 4.5 (*t*(33) = 29.946, *p* < 0.001; Cohen's *d* = 5.212, 95% CI [4.105, Inf]) and desirability ratings of adjectives classified as negative showed to be significantly below 4.5 *t*(33) = −13.993, *p* < 0.001; *d* = −2.435, 95% CI [−Inf, −1.856]). For studies 2, 3, and 4 we selected and screened 120 adjectives with a separate sample (*n* = 82) according to different criteria (see, Stimuli, Study 2).

For all the experiments, we report how we determined our sample sizes, all data exclusions, all manipulations, and all measures in the study. Study materials are available at Data S1. Data and analysis code are available at (https://osf.io/yeg8v/?view_only=2c92126b0b1344c0a1ee84cedc3ee482). Data were analyzed using R, version 4.2.2 (R Core Team 2020). This study's design and its analysis were not preregistered.

### 2.3 Results

Following prior research, we first assessed whether participants updated their self‐representations more in response to positive than negative feedback. To that end, we conducted a repeated measures Analysis of Variance (rmANOVA) with average update scores as the dependent variable, Feedback valence (positive vs. negative) as a within‐participants factor, and feedback discrepancy and update space as covariates. Results showed that participants tended to update their beliefs more in response to positive than to negative feedback (positive feedback: *M* = 0.376, SE = 0.109, 95% CI [0.157, 0.549]; negative feedback: *M* = −0.195, SE = 0.109, 95% CI [−0.414, 0.023], *F*(1, 30) = 12.004, *p* = 0.001, *η*_p_^2^ = 0.285, 90% CI [0.076, 0.461]). These results replicated the valence‐dependent belief updating effect described in previous literature.

Next, we quantified how participants' initial self‐evaluations masked the effect of feedback valence and feedback self‐congruence on participants' self‐concept updating. We assumed participants self‐concepts to be positively biased, that is, they would make more positive than negative categorical decisions about their attributes. This initial positive bias creates a scenario where most positive feedback overlaps with feedback that aligns with the individual's self‐views (self‐congruent feedback), and likewise, most negative feedback overlaps with feedback that conflicts with their current self‐concept (self‐incongruent feedback). This potential overlap would obscure the distinct effects of feedback valence and feedback self‐congruence. In the most extreme scenario, a participant who only makes positive categorical self‐judgments would find positive feedback 100% self‐congruent (confirming their notion that all positive traits are self‐descriptive, and all negative traits are not). In contrast, they would find all negative feedback incongruent, as it would challenge their self‐concept in every trial. To quantify this overlap, we first tried to predict participants' categorical judgments (yes vs. no) using adjective valence (positive vs. negative) as a predictor, by means of a mixed‐effects logistic regression (participants' ID as a random effect). The results of this analysis showed that adjective valence strongly predicted participants' categorical self‐judgments (Marginal *R* ^2^: 0.383, Conditional *R* ^2^: 0.391). Specifically, we found that it was 17.727 times more likely to choose a positive than a negative adjective as self‐descriptive (OR_positive_: 17.727, SE = 3.263, 95% CI [12.358, 25.430], *z* = 15.618, *p* < 0.001).

Next, we aimed to quantify to which extent a positive feedback trial would be also classified as a congruent feedback trial, which provides a measure of the overlap between conditions. The results of this analysis suggested that a positive feedback trial was 11.049 times more likely to be classified also as a congruent than a negative feedback trial (OR_positive_: 11.049, SE = 1.634, 95% CI [8.268, 14.764], *z* = 16.242, *p* < 0.001). Our results, therefore, suggested that the belief updating bias attributed to the effect of feedback valence could be similarly ascribed to feedback's congruence with participants' initial self‐concept.

Finally, we compared updating scores, averaging them across feedback self‐congruence categories (self‐congruent vs. self‐incongruent) instead of across feedback valence conditions. Analogously to the results obtained with feedback valence, the results suggested that participants tended to update their beliefs more in response to self‐congruent feedback than to self‐incongruent feedback (self‐congruent feedback: *M* = 0.617 SE = 0.175 95% CI [0.266, 0.968]; self‐incongruent feedback: *M* = −0.456 SE = 0.175 95% CI [−0.807,0.105], *F*(1, 30) = 7.484, *p* = 0.011, *η*_p_^2^ = 0.199, 90% CI [0.028, 0.382]).

### 2.4 Discussion

In experiment 1, we showed that to appropriately evaluate the possibility that our self‐representations are updated in a valence‐dependent manner, we should first disambiguate the effect of feedback valence from feedback self‐congruence. To do so, we should first address participants' positive bias in self‐concept, as it produces an overlap between positive‐congruent and negative‐incongruent feedback trials.

The discussion regarding the degree to which individuals emphasize self‐concept positivity as opposed to stability has been a longstanding topic in the literature (Kwang and Swann 2010). New research has offered a more nuanced perspective on positivity constraints on self‐concept updating by examining how individuals update their self‐concept in response to self‐relevant feedback. This research has shown that we not only strive to receive positive feedback and seek out those who give it to us (Alicke and Sedikides 2009; Hepper et al. 2010; Pyszczynski et al. 1985) but, in fact, we asymmetrically incorporate positive vs. negative self‐relevant feedback to increase our self‐concept positivity (Elder et al. 2022; Korn et al. 2012, 2014, 2016). Although this research has provided robust results and large effect sizes, it mainly focuses on studying how positive feedback selectively induces self‐concept update (but see, Elder et al. 2022). This approach overlooks the potential of that feedback to reinforce or conflict with the current self‐concept, thereby enhancing or challenging self‐concept stability. Our results suggest that existing evidence cannot conclusively attribute the observed biases in self‐concept updating solely to the pursuit of self‐concept positivity. Indeed, they might be similarly explained by our need to maintain a stable self‐view.

The orthogonalization of feedback self‐congruence and feedback valence would allow us to study at the same time whether motives for positivity and stability drive the integration of social evaluative feedback in self‐concept representations.

## 3 Experiment 2

### 3.1 Introduction

The findings from Experiment 1 highlighted the importance of distinguishing between positivity‐seeking and stability‐seeking influences when studying the incorporation of feedback into the self‐concept. To accurately disentangle these effects, it is necessary to control for potential positive biases in participants' self‐concept. The majority of the population tends to have a positively biased self‐concept (Alicke and Sedikides 2009; Taylor et al. 1988). However, it is common for individuals to recognize some negative aspects of themselves and acknowledge the absence of certain socially desirable traits they may wish to possess (Baranski et al. 2021; Higgins 1987; Steele 1988). We propose that an effective way to control for the effect of the positive bias in the self‐concept might be to sample individuals' beliefs about their traits. Our proposal involves utilizing a non‐proportional stratified random sampling method to gather participants' positive and negative decisions concerning their self‐concept. This approach would ensure that for every participant a balanced set of positive and negative decisions is available. Importantly, this approach could also be used to control the effect of both positively and negatively biased self‐concepts.

As outlined before, an exclusive focus on either seeking positivity or maintaining stability in our self‐concept could be suboptimal for our well‐being. Alternatively, both motives might have room to influence how we deal with self‐relevant feedback (Mokady and Reggev 2022; Swann et al. 1989). Our drive for stability in our self‐representations may result in the preferential incorporation of self‐congruent feedback. Its integration would add consistent evidence to an already well‐grounded self‐concept (Conway 2005; Rathbone et al. 2008), which would be neutral in terms of positivity gains (it would confirm both our positive and negative aspects, or lack thereof). Gaining self‐concept stability could in itself be beneficial for well‐being (Campbell 1990), but it could compromise progress toward a more positive self‐concept, as it might also imply adding confirming evidence on negative traits or our lack of desirable traits. If that is the case, we hypothesize that this could be solved by biasing the integration of self‐congruent feedback in favor of those evaluations that, besides being self‐congruent, are positive in nature. In turn, a drive for positivity may lead to an enhanced integration of positive feedback, leading us to see ourselves in a better light. However, the indiscriminate integration of positive feedback over negative feedback could be detrimental to the stability of the self‐concept. Specifically, it might involve ceasing to identify ourselves with negative attributes strongly supported by our autobiographical knowledge or starting to believe that we possess traits that do not reflect our behavioral patterns. This might lead to increased uncertainty about ourselves, our abilities, goals, or feelings (Conway 2005; Conway et al. 2004; Kim and Chiu 2011; Swann Jr. and Brooks 2012). Note that accepting negative self‐congruent feedback to the same extent as negative self‐incongruent feedback might also be suboptimal. Although we assume that negative social evaluations would be integrated to a lesser extent, the integration of congruent negative feedback would reinforce our certainty about our self‐concept, while the integration of self‐incongruent feedback would bring the double penalty of loss of positivity and stability.

In Experiment 2, we aimed to test the effect of both positivity and stability constraints on participants' integration of self‐relevant social feedback. We hypothesized that participants would incorporate more self‐congruent than self‐incongruent feedback into their self‐representations as well as more positive than negative feedback. Here, we also aimed to explore whether feedback‐induced changes in self‐representations would remain 1 day after the experiment. This test may provide further understanding of how self‐concept representations are shaped and maintained, along with their potential variability depending on the type of feedback received.

### 3.2 Methods

#### 3.2.1 Participants

For this experiment, we recruited 45 participants (Spanish undergraduate students, 29 female and 16 male, *M*_age_ = 22.19 years, SD_age_ = 2.11 years). The required sample size was obtained by another power analysis, which determined that to detect an effect of *η*_p_^2^ = 0.05 (power = 0.8, *α* = 0.05) we would need at least 28 participants. Although prior studies have found higher effect sizes, we decided to reduce the effect size parameter because we expected some of the explained variance attributed to feedback valence to be captured by feedback self‐congruence. Three participants were excluded from the sample based on their BDI‐II scores (participant 1: 21, participant 2: 23, participant 3: 23). One participant was excluded from the sample based on their number of missing responses [> 15%, (participant percentage of missing responses = 46.87%)]. The final sample was composed of 41 participants (28 female and 13 male *M*_age_ = 22.44 years, SD_age_ = 2.32 years).

#### 3.2.2 Procedure

Participants took part in a three‐session experiment on 3 consecutive days. The objective of the initial session was to set up a scenario where participants anticipated receiving relevant social feedback during the subsequent session. The second session consisted of performing the experimental tasks (see, Experiment 1). The aim of the third session was to obtain a follow‐up measure of the effects studied in Session 2. All participants were informed about the experimental manipulation right after the conclusion of Session 3.

As outlined above, to effectively investigate how the pursuit of maximizing positivity or stability influences the incorporation of information into the self‐concept, it is crucial to distinguish and analyze their individual effects. To accomplish this goal, the overlap that our positively biased self‐concept posits between positive and self‐congruent feedback as well as between negative and self‐incongruent feedback needs to be experimentally controlled. Here we introduced a novel approach to that end, namely, a non‐proportional stratified random sampling of participants' positive and negative decisions about the self. This process allows an unbiased part of the participants' self‐concept to be subjected to the belief updating task. The procedure was introduced in the sentence verification task (Figure 1A). As outlined in Study 1, this task involves participants making positive (rejecting negative traits or accepting positive traits) and negative (rejecting positive traits or accepting negative traits) self‐relevant decisions about various traits. We used an extended list of adjectives (*n* = 95, see below, Stimuli) to increase the number of decisions participants would make about themselves. The non‐proportional stratified random sampling consists of a random selection of the same percentage of elements in all strata (here positive and negative decisions) to achieve a balanced sample. In the sentence verification task, participants judged the 95 adjectives in random order and provided confidence judgments for all decisions. From all the (mostly positive) decisions made, a minimum number of them per stratum was extracted. That is, the same number of positive and negative decisions were randomly drawn from their respective populations. Based on the data provided by Study 1, we estimated that the percentage of positive decisions participants would make would be between 80% and 82% of the total (Study 1 upper bound 99% CI = 81.6%) which would imply making between 77 and 79 positive decisions out of 96 adjectives in the most extreme cases. To obtain balanced conditions (positive and congruent feedback, positive and incongruent feedback, negative and congruent feedback, and negative and incongruent feedback) half of the positive decisions should receive negative feedback and the other half positive feedback, and the same for negative decisions. Therefore, we set at 16(/96) the minimum number of negative decisions necessary for a participant to be included in the analysis. One participant did not meet this inclusion criterion and was excluded from further analysis (effective sample size: *n* = 40). Once this process was completed and a balanced set of positive and negative decisions was reached, participants underwent the belief updating task.

Participants were also tested 1 day after the completion of both tasks to test the potential long‐lasting effects of feedback valence and feedback congruence on participants' self‐representations. In this session, participants provided again categorical decisions about their own traits. Judgments only included those participants' decisions that were filtered in session two by the non‐proportional stratified random sampling. Next, participants provided self‐ratings (Likert scale from 1 to 8) on the same traits, as in the first part of the belief updating task.

##### 3.2.2.1 Stimuli

The non‐proportional stratified random sampling of participants' positive and negative decisions required us to extend the list of adjectives used in the pilot study. To this end, we selected an initial sample of 120 adjectives drawn from previous studies (Anderson 1968; Dumas et al. 2002; Korn et al. 2012, 2016) on the basis of their valence (60 positive and 60 negatives). To further refine the list of adjectives, we aimed to obtain measures of valence and familiarity from a separate sample of participants with similar characteristics (age and educational level) to the one that would carry out our experiment. We recruited a sample of 82 university students (57 females and 25 male, Age: *M* = 21.57, SD = 1.64). Participants judged adjectives' valence and familiarity using a Likert scale ranging from 1 to 8. Since our experiments involved participants evaluating other people on the basis of personality descriptions and believing that other people would evaluate them, we also wanted to select those adjectives that were judged to be ‘most observable’ in this context. Thus, as in the separate sample we recruited for the same purpose in Experiment 1, we asked participants to assess the extent to which they considered each adjective to be ‘observable’ by listening to a recording of a 6‐min personality description. We provided each participant with the guidelines that participants in our experiment were to use to make their recordings. To filter the final sample of adjectives, we selected as positive adjectives those with an average score equal to or higher than 5 (on a scale from 1 to 8), and as negative adjectives those with a score equal to or lower than 4. In addition to this criterion, only those adjectives with an average familiarity and observability score above 4.5 were selected. The final set of stimuli consisted of 49 positive and 47 negative adjectives (see Table S3).

### 3.3 Results

To test the effect of both feedback valence and feedback self‐congruence on participants' belief updating, we conducted a rmANOVA with average updating scores as the dependent variable, feedback valence, feedback self‐congruence, and their two‐way interaction as within‐participants factors, and Feedback discrepancy and Update space as covariates. Estimated marginal means (Lenth 2022) are reported. For non‐significant results associated with the hypotheses (main effects), bayes factors in favor of the alternative hypothesis are reported. Results showed that participants tended to update significantly more their self‐representations in response to Self‐congruent feedback than in response to Self‐incongruent feedback (self‐congruent: *M* = 0.472, SE = 0.133, 95% CI [0.210, 0.733], self‐incongruent *M* = −0.131, SE = 0.133, 95% CI [−0.393, −0.131], *F*(1, 37) = 14.081, *p* < 0.001, *η*_p_^2^ = 0.28, 90% CI [0.091, 0.442]). Results also showed a non‐significant main effect of feedback valence (positive feedback: *M* = 0.112, SE = 0.061, 95% CI [0.107, 0.348], negative feedback *M* = 0.228, SE = 0.061, 95% CI [−0.008, 0.233], *F*(1, 37) = 0.276, *p* = 0.603, *η*_p_^2^ = 0.007, 90% CI [0, 0.051], BF_10_ = 0.102) and a non‐significant feedback self‐congruence × feedback valence interaction (*F*(1, 37) = 0.048, *p* = 0.828, *η*_p_^2^ = 0.001, 90% CI [0, 0.018]).

As suggested by prior studies (Garcia‐Arch et al. 2022; Korn et al. 2012), we explored the distribution of feedback discrepancies among the different experimental conditions. We conducted a rmANOVA with average feedback discrepancy as the dependent variable and feedback self‐congruence, feedback valence, and feedback self‐congruence × feedback valence interaction as within‐participants factors. We found a significant main effect of feedback valence (*F*(1, 39) = 18.959, *p* < 0.001, *η*_p_^2^ = 0.33, 90% CI [0.134, 0.483]), a significant main effect of feedback self‐congruence (*F*(1, 39) = 456.901, *p* < 0.001, *η*_p_^2^ = 0.92, 90% CI [0.873, 0.940]), and a significant feedback self‐congruence × feedback valence interaction (*F*(1, 39) = 7.054, *p* = 0.011, *η*_p_^2^ = 0.15, 90% CI [0.018, 0.308]). Post hoc contrasts (Estimated Marginal Means, Tukey's *p* adjustment) revealed that all self‐incongruent conditions received significantly larger feedback discrepancies than self‐congruent conditions. Results also showed that the self‐congruent + negative feedback condition was associated with higher feedback discrepancies than the self‐congruent + positive feedback condition (all *p* < 0. 001). These results suggest that our experimental conditions were strongly biased in terms of the feedback discrepancies they received.

### 3.4 Discussion

In Experiment 2, we aimed to examine how both the motivation for a positive self‐concept and the motivation for a stable self‐concept influence participants' incorporation of self‐relevant social feedback. We employed a method that allowed us to control the effect of participants' positively biased self‐concept and to generate balanced experimental conditions in which participants received self‐congruent, self‐incongruent, positive, and negative social evaluations. We hypothesized that participants would incorporate more self‐congruent than self‐incongruent feedback into their self‐representations as well as more positive than negative feedback. Our results showed that participants integrated substantially more self‐congruent than self‐incongruent feedback into their self‐representations by adjusting their self‐ratings in a self‐congruent feedback‐consistent direction. These findings are in line with models that suggest that the main motivation for our self‐concept is to be socially verified, which allows us to preserve identity stability and reinforces our perceived accuracy in our self‐judgments (Swann and Hill 1982; Swann, Stein‐Seroussi, et al. 1992) but extend them by indicating that the pursuit of stability constrains the capacity of social evaluative inputs to alter self‐representations.

Interestingly, feedback valence showed no effect in participants' updating of self‐representations. However, upon further examination of the data, it was observed that a key variable influencing belief updating, namely, feedback discrepancy (Korn et al. 2012), was not evenly distributed across the different feedback categories. Although its effects were statistically controlled for in our analysis, it is possible that the systematic differences in feedback discrepancies found across feedback conditions might have biased participants' behavior. Receiving feedback ratings more different from their own self‐assessments in the self‐incongruent conditions might lead participants to perceive self‐incongruent feedback even more self‐incongruent, leading to its detrimental integration. However, the opposite interpretation is also possible, that is, these larger feedback discrepancies might have heightened its integration and reduced the incorporation of the self‐congruent feedback, which may have been integrated to a greater extent if the feedback discrepancies had been evenly distributed across all conditions. In turn, the lack of a feedback valence effect could be partly explained by the fact that participants received larger feedback discrepancies in the self‐congruent negative feedback than in the self‐congruent positive feedback condition.

Any of these or other possible interpretations suggest that beyond statistical control, feedback discrepancies should be controlled experimentally. In fact, as in previous studies (see, Procedure, Experiment 1), feedback discrepancies were pseudo‐randomly generated so that half the time they resulted in positive feedback and the other half in negative feedback. This manipulation generates conditions with similar feedback discrepancies between conditions when these are split into positive and negative feedback (Garcia‐Arch et al. 2022; Korn et al. 2012). However, this manipulation does not take into account self‐congruence. In the self‐congruent feedback conditions, participants receive feedback that is ‘confirmatory’. This means that for participants who judge the trait “Sociable” as non‐representative and assign themselves a 3 in their self‐rating, the feedback they will receive can only be lower than 3. In this same example, if this trait were in the self‐incongruent condition, the feedback received could take any value above 3 and up to 8. The randomization of feedback discrepancies is consequently biased between the two conditions because its values are sampled from unequal value ranges. We addressed this issue in Study 3 along with additional considerations.

## 4 Experiment 3

### 4.1 Introduction

Findings from Experiment 2 revealed that only social feedback that confirms our self‐concept seems to be integrated into self‐representations. However, upon closer analysis of the data, we observed a notable imbalance in feedback discrepancies among the different conditions, which might have influenced the results. Thus, our aim was to make methodological adjustments to enhance the robustness of the evaluation of the main hypotheses in Experiment 2.

### 4.2 Methods

#### 4.2.1 Participants

For this experiment, we recruited 52 participants (see Participants Experiment 2, for details on the power analysis). Two participants were excluded from the sample based on their BDI‐II scores (score participant 1: 32, score participant 2: 24). One participant was excluded from the sample based on their number of missing responses [> 15% (% missing responses: 90%)]. As in Experiment 2, we also excluded from the sample those participants that did not make a minimum number of negative decisions about their self‐concept. This procedure excluded only one participant in Experiment 2, where the minimum number of negative decisions was set to 16/96. Given the low exclusion rate in Experiment 2, we decided to increase the total number of trials of the experiment and the required number of negative decisions accordingly (20/96). Four participants did not reach the minimum number of negative decisions required and were excluded from the sample. The final sample was composed of 45 participants (Spanish undergraduate students, 32 female and 23 male *M*_age_ = 21.76 years, SD_age_ = 1.39 years).

#### 4.2.2 Procedure

The procedure employed in this study was the same as in Experiment 2, with two modifications. First, to avoid asymmetries in the distribution of feedback discrepancies across feedback conditions, we restricted the range of values among which feedback was sampled to be the same in all conditions. We adjusted this range based on the condition with lower average feedback discrepancies according to Study 2. Feedback values were computed by adding or subtracting values from 1 to 15 to participants self‐ratings (see, Methods, Study 1, for details). Second, and in line with the same aim, we changed the 8 points Likert scale to a scale with a wider range of values (1–100), which has been also used in belief updating paradigms (Sharot and Garrett 2016). The main reason to consider the 8 points Likert scale problematic was that it generates a range restriction in feedback values and possible updates. The limited 8‐point range for self‐ratings used in prior studies (Korn et al. 2012, 2014, 2016), as well as in our Experiments 1 and 2, has the limitation that in some scenarios it may influence the potential updating of participants' beliefs. To exemplify one of its limitations, imagine that a participant provides a self‐rating of 5 for the adjective ‘sociable’. If this participant receives a 7 as a feedback rating, they could update their belief in a feedback‐consistent direction by moving it up to the feedback value offered. This scenario already entails a problem, as it would only allow counting as updates those belief changes that represent a percentual change of at least 12.5% (increments or decrements of 1 over the total scale), which could imply a loss of belief updating sensitivity. A more problematic case might arise when the feedback received only implies a discrepancy of (+/−) 0.5, or (+/−) 1. In these cases, belief updating could not occur within the range between the initial belief and the feedback received, since self‐assessments are limited by the 8‐point Likert scale. Changing to a wider (1–100) scale has the potential to solve both problems by providing sufficient range of values for both self and feedback ratings.

### 4.3 Results

In this study, we aimed to solve the asymmetry in feedback discrepancies across feedback conditions found in Experiment 2 to provide a more valid test for our hypotheses. To check if our methodological adjustments managed to cancel out the asymmetry in feedback discrepancies across feedback conditions, we conducted a rmANOVA with average feedback discrepancy as the dependent variable, and feedback self‐congruence and feedback valence as within‐participants factors. Results showed no significant feedback discrepancy asymmetries across feedback conditions (feedback self‐congruence *F*(1, 44) = 0.151, *p* = 0.699, *η*_p_^2^ = 0.003, 90% CI [0, 0.030], feedback valence *F*(1, 44) = 0.011, *p* = 0.919, *η*_p_^2^ < 0.001, 90% CI [0, 0.007], feedback self‐congruence × feedback valence *F*(1, 44) = 0.067, *p* = 0.798, *η*_p_^2^ = 0.001, 90% CI [0, 0.016]). While further refinements (such as adaptive feedback matching procedures) might be explored in future research, we believe these improvements sufficiently address the imbalance noted in Experiment 2.

**FIGURE 2:**
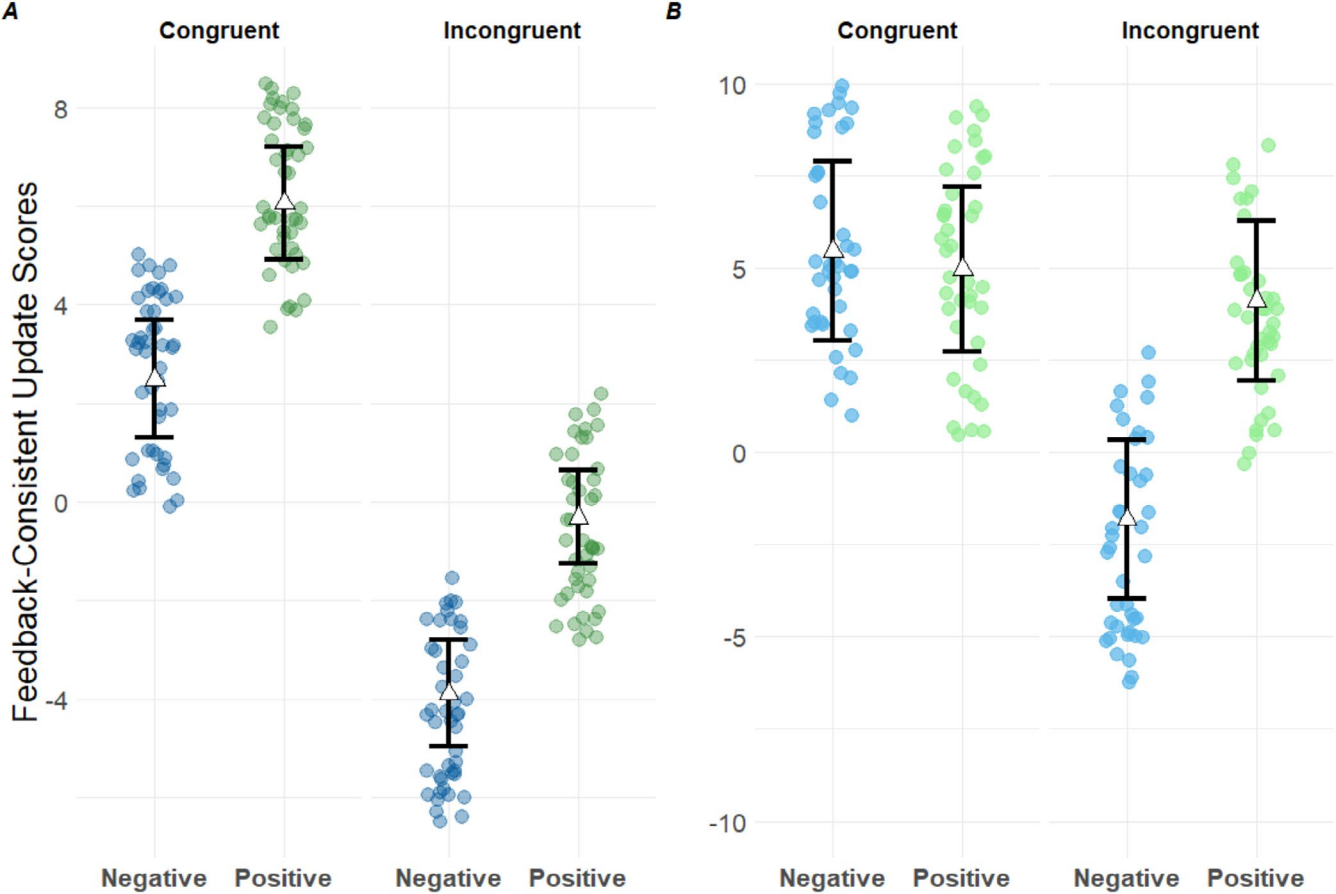
Differences in Feedback‐consistent belief update scores between feedback self‐congruence (facet) and feedback valence conditions (colors) in Experiments 3 (A) and 4 (B). Triangles represent the estimated marginal means of each feedback condition (Lenth 2022), points represent a sample of model residuals in each condition; the 95% confidence interval is indicated by the ends of the vertical error bar.

After testing the effectiveness of the experimental control on feedback discrepancies, we set out to reproduce the main analysis carried out in Experiment 2. We conducted a rmANOVA with average update scores as the dependent variable, feedback valence, feedback self‐congruence, and their two‐way interaction as within‐participants factors, and feedback discrepancy and update space as covariates. Results showed a significant and large main effect of feedback self‐congruence (*F*(1, 42) = 16.250, *p* = < 0.001, *η*_p_^2^ = 0.278, 90% CI [0.098, 0.431]), a significant and medium effect of feedback valence (*F*(1, 42) = 4.537, *p* = 0.039, *η*_p_^2^ = 0.097, 90% CI [0.002, 0.231]) and no significant feedback self‐congruence × feedback valence interaction (*F*(1, 42) = 2.975, *p* = 0.091, *η*_p_^2^ = 0.061, 90% CI [0, 0.172]). Next, we aimed to conduct a more fine‐grained analysis by means of linear mixed‐effects models (LMMs). This approach allows us to capture and account for individual differences in the effects tested, provide interpretable estimates for any term of the model, compute proper marginal effects and post hoc tests, and incorporate additional random effects in the covariance structure of the model tested (Baayen et al. 2008; Barr et al. 2013; Brown 2021). To obtain the best LMM, we constructed alternative models that varied in their inclusion of random intercepts and slopes and compared them by means of the Bayesian Information Criteria (BIC), which penalizes model complexity (Schwarz 1978). *p*‐values were determined by Satterthwaite's approximation of degrees of freedom (Kuznetsova et al. 2017). For model construction, we started with the LMM version of our rmANOVA model, which included fixed effects for feedback self‐congruence, feedback valence, feedback discrepancy and update space, and participants' ID as a grouping factor. We subsequently tested if this model could be improved by sequentially adding different combinations of: partially crossed random effects (Adjectives and Participants' ID), the interaction between feedback self‐congruence and feedback valence, and random slopes for feedback self‐congruence and feedback valence. Maximal random effects structures were kept when supported by the data and model convergence (Barr et al. 2013). Among all LMMs, only one outperformed the starting model. This model included fixed effects for feedback self‐congruence, feedback valence, feedback discrepancy and update space, participants' ID as a grouping factor, and a random slope for feedback valence (Maringal *R* ^2^ = 0.166, Conditional *R* ^2^ = 0.204). The LMM converged with the results obtained by the rmANOVA. Results showed that participants tended to update significantly more their self‐representations in response to self‐congruent feedback than in response to self‐incongruent feedback (*β*_Self‐congruent_ = 6.426, SE = 0.699, 95% CI [5.053, 7.798], *t*(1701.380) = 9.185, *p* < 0.001) and in response to positive (vs. negative) feedback (*β*_positive_ = 3.591, SE = 0.731, 95% CI [2.156, 5.025], *t*(46.183) = 4.911, *p* < 0.001; Figure 2A). Results also showed no significant relationship between feedback discrepancy and update scores (*β* = −0.031, SE = 0.072, 95% CI [−0.172, 0.111], *t*(1733.021) = −0.427, *p* = 0.669) and a positive and significant relationship between update space and update scores (*β* = 0.213, SE = 0.012, 95% CI [0.189, 0.237], *t*(1722.983) = 17.425, *p* < 0.001). To further explore differences in feedback‐consistent belief updating across feedback conditions, we computed Sidak adjusted confidence intervals (Cis) on the marginal means (Lenth 2022). The analysis showed that participants integrated social evaluative feedback in their self‐representations (adjusted CIs did not include 0) in both self‐congruent feedback conditions (positive feedback: *M* = 6.121, SE = 0.628, 95% CI [4.531, 7.711], negative feedback: *M* = 2.531, SE = 0.553, 95% CI [1.122, 3.937]). Results also revealed that self‐incongruent and positive feedback did not affect participants self‐representations (*M* = −0.305, SE = 0.578, 95% CI [−1.777, 1.167]) while self‐incongruent and negative feedback tended to be rejected, that is, participants tended to update their self‐representations in the opposite direction (*M* = −3.896, SE = 0.608, 95% CI [−5.433, −2.359]).

Next, we conducted an exploratory analysis to test whether the observed effects remained 1 day after the experimental Session. 5 (11%) participants did not participate in the follow‐up test. We conducted a rmANOVA with update scores as the dependent variable, feedback self‐congruence, feedback valence, time (Session 2, follow‐up) and their three‐way interaction as within‐participants factors, and feedback discrepancy and update space as covariates. Results showed a significant main effect of feedback self‐congruence (*F*(1, 37) = 14.755, *p* < 0.001, *η*_p_^2^ = 0.285, 90% CI [0.094, 0.447]) and a significant main effect of feedback valence (*F*(1, 37) = 5.188, *p* = 0.028, *η*_p_^2^ = 0.123, 90% CI [0.007, 0.276]). No significant main effects were found for time (*F*(1, 39) = 0.059, *p* = 0.809, *η*_p_^2^ = 0.001, 90% CI [0, 0.017]), feedback self‐congruence × feedback valence interaction (*F*(1, 37) = 0.924, *p* = 0.342, *η*_p_^2^ = 0.024, 90% CI [0, 0.103]), feedback self‐congruence × time interaction (*F*(1, 39) = 0.157, *p* = 0.694, *η*_p_^2^ = 0.004, 90% CI [0, 0.037]), feedback valence × time interaction (*F*(1, 39) = 0.423, *p* = 0.519, *η*_p_^2^ = 0.017, 90% CI [0, 0.083]), or feedback self‐congruence × feedback valence × time interaction (*F*(1, 39) = 0.685, *p* = 0.413, *η*_p_^2^ = 0.017, 90% CI [0, 0.083]).

### 4.4 Discussion

In Experiment 3, we addressed methodological limitations of Experiment 2 to provide a more reliable testing ground for our hypotheses. Our methodological adjustments successfully eliminated feedback discrepancy asymmetries across the different feedback conditions. Our findings suggested that both our need to gain self‐concept positivity and our need to maintain self‐concept stability play a role in constraining the integration of self‐relevant social feedback.

The primary analyses of Study 3 confirmed the significant effect of feedback self‐congruence, mirroring the results of Study 2. This consistency across studies supports our hypothesis that when processing self‐relevant feedback, individuals preferentially integrate information that reinforces their current self‐concept. Conversely, social inputs that conflict with our self‐views seemingly fail to stimulate a feedback‐contingent shift in our self‐representations, regardless of their valence. Contrary to the non‐significant effect of feedback valence in Experiment 2, a moderate effect was observed in Experiment 3. We found that positive feedback was moderately more integrated than negative feedback. A closer examination of the results suggested that not only did feedback valence have a lesser impact on participants self‐representations than feedback self‐congruence, but also greater variability among individuals. Interestingly, the examination of specific update scores under each condition suggested that only self‐congruent positive and negative feedback truly stimulated contingent changes in participants self‐representations. In contrast, self‐incongruent positive feedback seemed to have no effect, while self‐incongruent negative feedback involved substantial and directionally inconsistent updates of participants self‐representations. As this latter analysis was exploratory in nature, we cannot rule out the possibility that subtler update patterns, diverging from zero, might emerge if the sample size were increased. Further research could enrich our understanding of these patterns, complementing the initial findings presented here.

## 5 Experiment 4

### 5.1 Introduction

Experiment 3 suggested that existing self‐concept content plays a critical role in the incorporation of new self‐relevant information. Results revealed that individuals tend to preferentially integrate information that aligns with their pre‐existing self‐concept, and that this effect is more pronounced when the information received has the potential to improve self‐concept positivity. Although these results reconcile previous conflicting findings (Alicke and Sedikides 2009; Hepper et al. 2011; Swann Jr. and Brooks 2012; Swann, Stein‐Seroussi, et al. 1992), they do not provide any insights into whether the observed effects are specific to self‐concept or reflect a more general tendency to form and update beliefs about personality traits, including those of other people. This comparison (self vs. others) has been used as a strategy in a wide range of studies to provide insights into the functioning of our cognition, behavior, and affect, and represents a solid approach to test the influence of our self‐concept on many cognitive and perceptual domains (Chakraborty and Chakrabarti 2018; D'Argembeau and Van der Linden 2008; Frings and Wentura 2014; Jenkins and Mitchell 2011; Ma and Han 2010; Miles et al. 2010). In turn, the study of differences and similarities in the potential of congruent and incongruent feedback to generate changes in the representations we have about ourselves and others may be critical for understanding the formation and stabilization of our beliefs, and help clarify the boundaries between self‐related and social cognition.

In our daily experience, we constantly encounter new people and interact with them. To effectively navigate our social environments, we should form quick and accurate representations of others' personalities, which involves integrating information about their attributes during the belief formation process (Frolichs et al. 2022). There is also evidence of an asymmetry in favor of positive feedback when integrating information about the traits of others (Korn et al. 2012, 2014, 2016). However, it has been suggested that the mechanisms for updating beliefs about oneself and others are not identical. Although both self‐ and other‐focused belief updating may show a similar preference for positive feedback, the cognitive processes driving these updates might vary depending on whether the focus is on the self or someone else (Korn et al. 2012, 2014).

Critically, differences and similarities in belief updating patterns between the self and others may also emerge as a result of various combinations of the integration of congruent and incongruent feedback. When facing self‐related social feedback, the social inputs susceptible to be integrated into the self have to find their place in a well‐grounded self‐knowledge base, which contains autobiographical evidence supporting our self‐representations (Conway 2005; Haslam et al. 2011). According to our results, any information that challenges our self‐views might be rejected or ignored. In contrast, forming beliefs about others' personalities involves making judgments about their traits based on limited behavioral samples, which may entail a more dynamic process in which initially incongruent information is integrated to form a more precise (though possibly biased) view of others' attributes (Frolichs et al. 2022).

The purpose of this study was to determine the specificity of the effects observed in Experiment 3. We hypothesized that individuals would tend to incorporate more positive than negative feedback into their beliefs when evaluating the attributes of others. We also anticipated observing a diminished congruence effect, suggesting that individuals may exhibit a decreased resistance to modifying their pre‐existing beliefs when updating their perceptions about other people.

### 5.2 Methods

#### 5.2.1 Participants

For this study, we recruited 44 participants. To determine the sample size, we based the expected effect size (*η*_p_^2^) on prior research in this field, which has reported partial eta squared values above *η*_p_^2^ = 0.3 (e.g., Korn et al. 2012). As in our previous experiments, we decided to conduct a more conservative power analysis, assuming that part of the valence‐dependent belief updating effect might be captured by feedback congruence (see Participants Experiment 2 for details). Two participants were excluded from the sample based on their BDI‐II scores (score participant 1: 35, score participant 2: 28). One participant was excluded from the sample based on their number of missing responses [> 15% (% missing responses: 75%)]. As in experiments two and three, we also excluded from the sample those participants that did not make a minimum number of negative decisions in the sentence verification task. Two participants did not reach the minimum number of negative decisions required and were excluded from further analysis. The final sample was composed of 40 participants (Spanish undergraduate students, 28 female and 12 male *M*_age_ = 21.14 years, SD_age_ = 1.77 years).

#### 5.2.2 Procedure

In this study, we adapted the procedure previously used in Experiment 3. Participants listened to recordings of personality descriptions allegedly coming from former participants in the experiment. These recordings, which were approximately 7 min in length (*M* = 6.87, SD = 0.44), were made by 7 external collaborators (4 females, 3 males) who were unaware of the purpose of the study in order to maintain authenticity in their descriptions. Recordings were randomized across participants. After listening to the description, participants had to judge how similar they perceived their personality to be to the personality of the person of the recording using a scale from 1 (completely different) to 100 (identical). Participants then completed the sentence verification task, in which they provided dichotomous responses regarding the fit of 95 traits with the personality of the person described in the recording. Using non‐proportional stratified random sampling, the program obtained a balanced set of positive and negative categorical decisions. Finally, participants completed the belief updating task, which followed the same structure as in Experiments 2 and 3, with one variation: they were told they would receive feedback from three individuals who had also rated the person in the recording. The sentence preceding feedback in Experiments 1, 2, and 3 “Others think you are:” was replaced with “Others think this person is:”, followed by each filtered adjective and the corresponding feedback score.

### 5.3 Results

As in Study 3, we first checked whether feedback discrepancies did not significantly differ across feedback conditions. We conducted a rmANOVA with average feedback discrepancy as the dependent variable and Feedback congruence and Feedback valence as within‐participants factors. Results showed no significant feedback discrepancy asymmetries across feedback conditions (feedback self‐congruence *F*(1, 39) = 1.327, *p* = 0.256, *η*_p_^2^ = 0.032, 90% CI [0, 0.120], feedback valence *F*(1, 39) = 1.563, *p* = 0.219, *η*_p_^2^ = 0.038, 90% CI [0, 0.133], feedback self‐congruence × feedback valence *F*(1, 39) = 0.328, *p* = 0.571, *η*_*p*_^*2*^ = 0.008, 90% CI [0, 0.054]).

Next, we reproduced the analysis conducted in Experiments 2 and 3. We conducted a rmANOVA with average updating scores as the dependent variable, feedback valence, feedback congruence and their two‐way interaction as within‐participants factors, and feedback discrepancy and update space as covariates. Results showed a significant and moderate main effect of feedback self‐congruence (*F*(1, 37) = 5.964, *p* = 0.019, *η*_p_^2^ = 0.138, 90% CI [0.012, 0.296]), a non‐significant main effect of feedback valence (*F*(1, 37) = 0.201, *p* = 0.657, *η*_p_^2^ = 0.005, 90% CI [0.0, 0.042]) and a significant and strong interaction between feedback self‐congruence and feedback valence (*F*(1, 37) = 12.994, *p* < 0.001, *η*_p_^2^ = 0.259, 90% CI [0.080, 420]). Next, we followed the same analytical approach and conducted a similar analysis through LMMs. After model selection (BIC) the best model (Maringal *R* ^2^ = 0.113, Conditional *R* ^2^ = 0.164) included main effects for feedback congruence, feedback valence, feedback discrepancy and update space, an interaction between feedback self‐congruence and feedback valence, random intercepts for participants and a random slope for feedback valence. A global test for model predictors indicated that the interaction was statistically significant (*F*(1, 1472.69) = 15.849, *p* < 0.001).

To examine the interaction between feedback self‐congruence and feedback valence, we conducted pairwise comparisons with Tukey‐corrected *p*‐values and Sidak‐corrected confidence intervals. Our results showed that there were no significant differences in update scores among the congruent + positive feedback, congruent + negative feedback, and incongruent + positive feedback conditions (all *p* > 0.8). However, when receiving incongruent + negative feedback, participants updated their representations of others' traits significantly less compared to all other conditions (all *p* < 0.01). Sidak‐corrected CIs showed that participants integrated social evaluative feedback in their representations of others' traits (adjusted CIs did not include 0) when receiving both positive and negative self‐congruent feedback (positive feedback: 95% CI [2.107, 7.395], negative feedback: 95% CI [2.675, 8.061]) and when receiving incongruent + positive feedback (95% CI [1.659, 6.737]). However, incongruent + negative feedback did not alter participants beliefs (95% CI [−4.354, 1.212]).

In contrast to Experiment 3, the results of Experiment 4 suggested that changes in participants representations of others' traits where not fully restricted by the congruence of social evaluative feedback, as reflected by participants' integration of incongruent and positive information into their prior beliefs. Moreover, in Experiment 3, participants' bias toward positive feedback was reflected by the preferential and significant integration of positive (vs negative) congruent feedback, the dismissal of incongruent and positive feedback, and a substantial rejection of incongruent and negative feedback. However, in this study, the bias toward positive feedback only emerged in the incongruent feedback conditions, where negative feedback was dismissed and positive feedback was substantially integrated. To provide a direct comparison of results obtained in studies 3 and 4, we gathered the data and created a dichotomous variable representing the difference in the target under judgment (Experiment 3: Self, Experiment 4: Others).

We built a LMM that tried to capture the differences in the effects found between both studies. This model included main effects for feedback congruence, feedback valence, target (Self, Others), feedback discrepancy, update space, and a three‐way interaction between feedback congruence, feedback valence, and target. Following the marginality principle, two‐way interactions were also included. To see if any alternative model could account better for the data, we compared the fitted model against LMMs including different combinations of slopes for feedback congruence and feedback valence. BIC determined that the best model (Maringal *R* ^2^ = 0.144, Conditional *R* ^2^ = 0.219) was that including the three‐way interaction, a random intercept for participants, and random slopes for feedback congruence and feedback valence. A global test on model predictors indicated that the three‐way interaction was statistically significant (*F*(1,3041.5) = 6.952, *p* = 0. 008). As evidenced by the significant interaction, participants from Studies 3 and 4 differed in their updating patterns. To further investigate these differences, we conducted a post hoc analysis comparing both groups (target) across each feedback condition (Tukey‐corrected *p*‐values). Results revealed no significant differences in belief updating between groups under congruent and negative feedback (*t*(106) = 1.508, *p* = 0.134) or congruent and positive feedback (*t*(114) = −1.233, *p* = 0.220). However, we found that participants in the “Others” group incorporated significantly more social evaluative feedback into their prior representations under both incongruent and positive feedback (*t*(110) = 3.503, *p* < 0.001) and incongruent and negative feedback (*t*(117) = 2.804, *p* = 0.005).

### 5.4 Discussion

In Experiment 4, we investigated whether the observed effects when updating beliefs about the self (Experiment 3) would extend to updating beliefs about the personality of others. Our findings indicated that when forming beliefs about others' attributes we display a more flexible approach that allows conflicting information to alter our initial representations. Notably, we also found that participants tended to incorporate more positive than negative feedback about others, in line with previous research (Korn et al. 2012, 2014). However, this effect was only present under incongruent feedback. By examining condition‐specific update scores we found that all feedback types, except for incongruent and negative social inputs, elicited feedback‐consistent shifts in participants' prior beliefs. Interestingly, while incongruent and negative feedback led to substantial and directionally inconsistent updates in participants beliefs about the self, this same type of social inputs resulted in negligible updates in participants' beliefs about others. These novel findings shed light on potential self‐specific constraints on belief formation and change and suggest that we display belief updating patterns especially suited to maintain a positive and stable self‐concept.

The results of Experiment 4 also provide insights into how we form beliefs about other people's personalities. Prior research has shown that when facing new information, we also display a tendency to incorporate more positive than negative feedback about others. To our knowledge, this study is the first to experimentally control the initial positive bias in both our self‐concept and our beliefs about others' personalities and test the effect of feedback congruence. Importantly, our findings highlight that although a positivity bias is evident when updating representations of both the self and others, the specific patterns of integration of congruent and incongruent information might differ between the two. These findings contribute to a deeper understanding of belief updating processes and emphasize the distinct nature of self‐beliefs compared to beliefs about others' personalities.

## 6 General Discussion

In this research, we tested the idea that when updating our self‐representations, a balance exists between the need for adaptability in our self‐concept to foster positivity and the need for consistency to preserve our existing self‐knowledge. We conducted a comprehensive series of experiments focusing on the integration of new self‐relevant information. Our aim was to orthogonalize and test the influences of both feedback self‐congruence and feedback valence on the updating of self‐representations. Our findings revealed that both the need to preserve existing self‐knowledge and the desire for a positive self‐image impose self‐specific constraints on the integration of new self‐relevant information. By considering the simultaneous roles of positivity and stability motives, we gain a deeper understanding of how our self‐concept is shaped and maintained over time.

Our study suggests that when facing new self‐relevant social feedback, we tend to preferentially integrate information that aligns with our pre‐existing self‐concept. This is consistent with research suggesting that we strive for social verification of our self‐representations (Swann Jr. and Brooks 2012; Swann and Buhrmester 2012). Importantly, our findings go beyond existing evidence suggesting that we display a preference for receiving self‐congruent feedback and seeking like‐minded interaction partners and suggest that we actively use self‐congruent information to strengthen our self‐representations, potentially maximizing self‐concept stability. Our results also align with the notion that self‐representations are embedded within a robust system of self‐knowledge that helps us selectively process and assimilate new self‐congruent information (Conway 2005; Conway et al. 2004; Haslam et al. 2011; Klein 2010). Displaying asymmetric integration of self‐congruent versus self‐incongruent feedback might progressively help individuals distinguish between self‐descriptive and non‐self‐descriptive attributes and improve self‐concept clarity (Campbell 1990). This would have important implications for everyday functioning, as having a clear view of our own attributes helps us generate predictions about our future, plan our actions, select appropriate interaction partners, and preserve our psychological well‐being (Becht et al. 2017; Campbell 1990; Campbell et al. 2003; Lewandowski and Nardone 2012).

In line with prior research, our results suggested that when facing self‐relevant social feedback, we also tend to integrate more favorable than unfavorable information into our self‐concept (Korn et al. 2012). This effect draws on the notion that we are strongly motivated to pursue positive self‐representations, even at the expense of accuracy (Alicke and Sedikides 2009; Sedikides and Alicke 2019; Taylor et al. 1988). However, the inclusion and experimental orthogonalization of the effect of feedback self‐congruence in our study provide novel insights that challenge and refine existing research. Although both positivity and stability motives showed effects on participants' integration of social feedback, our results suggested that the need to preserve existing self‐concept might exert a stronger influence. Specifically, we observed larger effect sizes and reduced variability across participants in the effect of feedback self‐congruence compared to feedback valence. Our findings challenge the prevailing notion that the pursuit of positive self‐representations is a universal and pervasive motivation among psychologically healthy adults (Alicke and Sedikides 2009) and emphasize the importance of maintaining one's existing self‐concept. One possibility is that self‐concept stability not only provides robustness to our self‐views but is also necessary to add new information to our existing self‐knowledge structures (Emery et al. 2015). If the incorporation of positive information were prioritized over self‐concept stability, the incorporated information might be as easily dismissed as it was initially accepted, leading to inconsistent self‐representations. We suggest that the incorporation of any self‐incongruent (positive or negative) changes into the self‐concept might necessitate the presence of stability mechanisms (Conway 2005; Conway et al. 2004). These mechanisms might serve to preserve the structural integrity of the self‐concept during change processes and stabilize the newly acquired self‐knowledge.

Although participants prioritized both self‐congruent and positive feedback inputs over their counterparts (self‐incongruent and negative feedback), our analysis of condition‐specific update scores revealed that only self‐congruent (positive and negative) evaluations induced feedback‐consistent updates in participants' self‐representations. The results from the current research revealed a positivity bias within the self‐congruent feedback conditions, wherein participants exhibited a stronger inclination to modify their self‐representations in response to positive self‐congruent feedback compared to negative self‐congruent feedback. The same effect was found when examining update scores under self‐incongruent feedback levels. Interestingly, our results suggested that this positivity bias was driven by a lack of change under self‐incongruent and positive feedback and directionally inconsistent updates under self‐incongruent and negative feedback.

The specific updating patterns observed in our study offer novel insights that contribute to our understanding of the dynamics of stability and change in the self‐concept. To our knowledge, the lack of integration of positive self‐incongruent feedback about one's attributes has never been described. We propose that this phenomenon may be driven by the need to avoid self‐concept destabilization and maintain accurate predictions about our behaviors and affect (Conway 2005; Nowak et al. 2000; Steele 1988). Moreover, the feedback‐inconsistent updates observed in response to self‐incongruent and negative feedback may further reflect self‐concept protective mechanisms (Alicke and Sedikides 2009; Conway 2005; Nowak et al. 2000; Swann Jr. and Brooks 2012). Although self‐protection strategies have been widely described, previous studies have not differentiated the effects of feedback self‐congruence and feedback valence. This lack of distinction has resulted in overlapping predictions, suggesting that both negative feedback (whether congruent or incongruent) and incongruent feedback (whether positive or negative) would elicit compensatory reactions to protect our self‐views. Our findings indicate that these compensatory reactions may be specifically triggered by feedback that is both self‐incongruent and negative. We propose that the integration of negative social inputs that challenge our current self‐concept would impose a double penalty. First, it would involve destabilizing well‐grounded positive self‐representations or compel the integration of a new negative self‐representation. Both of these options have the potential to disrupt self‐concept stability (Conway 2005; Nowak et al. 2000). Besides, it would also lead to a reduction in the overall positivity of our self‐concept. In this context, our findings are consistent with the extensively described self‐protection strategies, but they provide further understanding of the specific circumstances under which these strategies might be triggered. However, since this finding was exploratory in nature, we cannot discard the existence of subtler effects. Future research should explore these specific updating patterns by targeting them as their primary analysis.

It is noteworthy that the feedback in our experiments was presented as coming from same‐aged peers. Given that young adults tend to place higher weight on feedback from similar and familiar individuals (Reitz et al. 2014), this aspect of our design may have amplified the observed effects on self‐concept updating. Future research should explore the role of feedback source characteristics by comparing responses to feedback from similar versus dissimilar individuals.

An important aspect of this research lies in the methodological refinement employed to orthogonalize the effect of stability and positivity motives on the integration of new self‐relevant information. This approach aligns with recent empirical and theoretical research that advocates for simultaneously considering both self‐concept motives in the study of self‐relevant feedback processing (Elder et al. 2022; Mokady and Reggev 2022). To the best of our knowledge, this is the first study to experimentally isolate these effects, which provides new strategies that might help refine existing findings. One potential example is the study from Korn et al. (2016) which investigated social feedback processing in patients with Borderline Personality Disorder (BPD). The researchers observed a reduced positivity bias in BPD patients compared to healthy controls, with the former showing a greater propensity to adjust their beliefs in response to negative feedback. Although this result is intuitively appealing, it could potentially be attributed to the tendency of BPD patients to maintain more negative self‐representations compared to healthy controls (Van Schie et al. 2020). This might lead them to perceive negative feedback as more consistent with their self‐concept. Moreover, BPD patients are not only characterized by more negative self‐views but also by a greater instability in their self‐concept (Kaufman and Meddaoui 2021). The methodological and analytical strategies we have developed could provide a valuable tool to quantify the influence of various aspects of their self‐concept on their reactions to social feedback, which might lead to the refinement of existing psychotherapeutic strategies. In addition, the approaches employed in this research might be useful to deepen our understanding of self‐relevant feedback processing, refining existing findings from behavioral and neuroimaging studies (e.g., Elder et al. 2022; Koban et al. 2023; Korn et al. 2012, 2014; Vanderhasselt et al. 2015; Yang et al. 2016).

Finally, our findings bridge the gap between two opposing perspectives on the primary motivations shaping our self‐concept (Kwang and Swann 2010; Sedikides and Alicke 2019). These seemingly opposing views have made similar claims on the importance of self‐concept stability and self‐concept positivity for our psychological well‐being. The current results not only suggest that both stability and positivity are important drivers in the development of our self‐concept, but they may also shed light on how the two are interconnected (Campbell 1990). By selectively integrating self‐congruent information, we might gain certainty and clarity about our own attributes, and the preferential integration of positive, self‐congruent information could serve as a mechanism for enhancing self‐concept positivity. We propose that individual differences in these feedback‐processing tendencies may accumulate over time, giving rise to distinct levels of self‐concept stability and self‐concept positivity in the population. This might shed light on potential mechanisms that underlie the relationship between important structural and affective components of the self (Campbell 1990; DeMarree and Rios 2014; Wong et al. 2016). In this sense, we hypothesize that these individual differences develop gradually, potentially over a timescale of several years—beginning in late adolescence and continuing into adulthood—as individuals repeatedly internalize social feedback. Future longitudinal research is needed to explore this dynamic process and to determine how it contributes to enduring differences in self‐concept stability and positivity.

This study is not exempt from limitations. In this work, we have implemented methodological improvements that have allowed us to examine how self‐concept gains stability and positivity through the selective integration of social feedback. However, our study has focused on the integration of social feedback about our own attributes (here, traits). Although this is a popular strategy and these personal representations are considered highly indicative of self‐concept, future research should broaden the scope to include other aspects of self‐concept, such as personal values or other forms of self‐relevant beliefs. For example, the belief updating patterns observed in this study would be particularly interesting to analyze in relation to physical self‐representation and core self‐evaluations (CSE), as both components play fundamental roles in self‐concept, mental health, and decision‐making processes (Benitez‐Sillero et al. 2024; Yudes et al. 2025). Examining belief updating through the lens of these self‐concept components could provide insights into individual differences in optimism bias, resistance to negative feedback, and the broader mechanisms of self‐concept maintenance. Study 4 revealed that the effects found might be self‐specific; however, this conclusion stems from a comparison (self vs. others) that involved gathering data from two distinct studies. Future research should rely on within‐subject designs or, alternatively, employ random assignment to self and other conditions. In addition, our sample was composed of young adults; the belief updating patterns found in this study might not be invariant across different age ranges or at different stages of self‐concept development. For example, there is some evidence that older adults exhibit larger valence‐dependent belief updating patterns (Chowdhury et al. 2014). Further research employing our orthogonalization of valence and self‐congruence should explore its potential variations across different age ranges. Similarly, although prior research has found no difference in valence‐dependent belief updating patterns across distinct cultural backgrounds (Korn et al. 2014), the orthogonalization of both effects could shed new light on cross‐cultural variations in self‐concept updating.

## 7 Conclusion

Understanding self‐concept dynamics is a complex endeavor that requires an effort to integrate knowledge and methodological approaches from different lines of research in psychology. Here, we aimed to contribute to the field by providing a more nuanced understanding of how our self‐concept is shaped and maintained by integrating past effort from social, personality, and cognitive psychology. By experimentally disentangling the effects of feedback self‐congruence and feedback valence, and leveraging insights from belief updating paradigms, we have shown that when facing new self‐relevant information, there is a trade‐off between stabilizing and enhancing our self‐views that might provide us with a progressively stable and positive self‐concept. It is important to clarify that our primary objective was not to articulate a fully developed new theoretical framework, but rather to empirically delineate the roles of stability and positivity in self‐concept updating. In doing so, we developed methodological and analytical strategies that can be applied to a wide range of studies in the domain of self‐concept and social feedback processing. We believe that this approach not only refines our current understanding of self‐concept dynamics but also lays the groundwork for future research aimed at developing a more comprehensive theoretical model.

## Author Contributions

All authors contributed to the conception and design of the study. Material preparation and data collection were carried out by Josué García‐Arch and Marc Sabio‐Albert. Josué García‐Arch performed the formal analysis and wrote the original draft of the manuscript. Lluís Fuentemilla provided funding, necessary resources, and supervised the project. All authors provided feedback on earlier drafts, read, and approved the final manuscript.

## Conflicts of Interest

The authors declare no conflicts of interest.

## Supporting information


**Data S1.** .

## Data Availability

Data and analysis code are available at https://osf.io/yeg8v/?view_only=2c92126b0b1344c0a1ee84cedc3ee482.
