# Supplementary material for: Selective Integration of Social Feedback Promotes a Stable and Positively Biased Self‐Concept
Source: Scand J Psychol. 2025 Apr 1;66(5):683–701. doi: 10.1111/sjop.13113 (PMC12423740; doi:10.1111/sjop.13113)
Supplement: Supplementary file 1 — Data S1. . [file SJOP-66-683-s001.docx]

**Supplementary Materials**

Please, find here the information referenced in the main text as *Supplementary Materials*. This file includes participants’ guidelines for audio recordings, and stimuli used in all the experiments.

| **Table S1.** Personality items provided for participants’ audio recordings |
| --- |
| **Items (Hexaco)** |
| If I knew that I could never get caught, I would be willing to steal a million euros. |
| I want people to know that I am an important person of high status. |
| I sometimes can't help worrying about little things. |
| Even in an emergency I wouldn’t feel like panicking. |
| I feel reasonably satisfied with myself overall. |
| I rarely express my opinion in social meetings |
| I am usually quite flexible in my opinions when people disagree with me. |
| I tend to be lenient in judging other people. |
| I prefer to do whatever comes to mind, rather than stick to a plan |
| People often joke with me about the messiness of my room or desk |
| I would be quite bored by a visit to an art gallery |
| People have often told me that I have a good imagination. |
| Note: Participants were instructed to talk about each statement referring to themselves. They were required to provide a simple answer and justify it by means of their personal opinion and experiences. |

| **Table S2.** List of adjectives (Experiment 1) | |
| --- | --- |
| **Adjectives** | **Valence** |
| Empathetic | Positive |
| Modest | Positive |
| Sociable | Positive |
| Enthusiastic | Positive |
| Generous | Positive |
| Creative | Positive |
| Honest | Positive |
| Astute | Positive |
| Cooperative | Positive |
| Respectful | Positive |
| Tolerant | Positive |
| Friendly | Positive |
| Polite | Positive |
| Organized | Positive |
| Composed | Positive |
| Aggresive | Negative |
| Arrogant | Negative |
| Superficial | Negative |
| Inapproachable | Negative |
| Impatient | Negative |
| Insecure | Negative |
| Stubborn | Negative |
| Anxious | Negative |
| Selfish | Negative |
| Obsessive | Negative |
| Chaotic | Negative |
| Wasteful | Negative |
| Moody | Negative |
| Cowardly | Negative |
| Lazy | Negative |

| **Table S3.** List of adjectives (Experiments 2, 3 and 4) | |
| --- | --- |
| **Positive** | **Negative** |
| Adventurous  Artistic  Astute  Attentive  Caring  Cheerful  Composed  Consistent  Cooperative  Courageous  Creative  Cultured  Convincing  Energetic  Empathetic  Enthusiastic  Friendly  Generous  Grateful  Honest  Humble  Imaginative  Independent  Ingenious  Insightful  Inspiring  Interesting  Loyal  Meticulous  Modest  Original  Optimistic  Patient  Philosophical  Pleasant  Polite  Practical  Persistent  Realistic  Reliable  Respectful  Sociable  Sincere  Spontaneous  Talented  Tolerant  Tough  Untiring  Warm | Aggressive  Anxious  Arrogant  Authoritative  Boring  Bossy  Careless  Chaotic  Childish  Cold-hearted  Conformist  Controlling  Cowardly  Forgetful  Gossipy  Gullible  Hostile  Immature  Impressionable  Inapproachable  Insecure  Irrational  Irritable  Lazy  Lonely  Materialistic  Melodramatic  Moody  Nervous  Noisy  Obsessive  Passive  Pessimistic  Possessive  Reckless  Rude  Sarcastic  Self-centered  Selfish  Serious  Strict  Stubborn  Superficial  Touchy  Unpunctual  Wasteful |
